# The balancing perspective of hard-to-reach hepatitis C patients who were lost to follow-up: A qualitative study

**DOI:** 10.1371/journal.pone.0230756

**Published:** 2020-04-13

**Authors:** Patricia A. M. Kracht, Joop E. Arends, Andy I. M. Hoepelman, Sigrid C. J. M. Vervoort

**Affiliations:** 1 Department of Internal Medicine and Infectious Diseases, University Medical Center Utrecht, Utrecht University, Utrecht, The Netherlands; 2 Cancer Center, University Medical Center Utrecht, Utrecht University, Utrecht, The Netherlands; Centers for Disease Control and Prevention, UNITED STATES

## Abstract

**Background:**

In the foreseeable future, patients with hepatitis C virus (HCV) with good healthcare access will all have been cured and the lost to follow-up (LFU) HCV-population will increasingly exist of hard-to-reach patients. Efforts to retrieve these individuals with HCV have been moderately successful so far. A deeper understanding of the reasons for loss to follow-up and the underlying processes is lacking.

**Aims:**

To explore reasons for previous loss to follow-up in patients with HCV who have been brought back into care.

**Methods:**

In 2017, fifteen patients with HCV who were evaluated at the University Medical Center Utrecht (UMCU) Infectious diseases outpatient clinic as part of the “REtrieval And cure of Chronic Hepatitis C” (REACH)-project were included in this study through convenience sampling. Face-to-face semi-structured in-depth interviews were conducted and a qualitative analysis based on the grounded theory was applied.

**Results:**

A basic socio- psychological process named “maintaining the achieved balance” was uncovered in patients with HCV who were LFU. This “achieved balance” is the result of a transformative process following the initial HCV diagnosis. It is a steadfast stance in which participants keep HCV out of sight and in the margin of their lives in order to reestablish an optimal state of well-being. The balancing perspective is subsequently defended by repeated evasive behavioral patterns to avoid confrontation with the disease.

**Conclusion:**

The balancing perspective gives insight into why individuals with HCV were not retained in care but also why they remained LFU thereafter. Physicians should realize that this mindset can be persistent and repeated efforts may be needed to finally trace and retrieve these patients.

## Introduction

Over the past few years, treatment possibilities of the hepatitis C virus (HCV) have reached their full potential with highly efficient and tolerable pangenotypic direct-acting antivirals (DAAs) now being available [1,2]. In order to achieve the World Health Organization (WHO) 2030 elimination targets [3], focus in the field of HCV healthcare has shifted towards increasing the diagnosis rate, improving linkage to care but also promoting adherence and preventing loss to follow-up [4]. Next to those undiagnosed, patients with HCV who are lost to follow-up (LFU) are an important source of untreated individuals with HCV [5].

Following several small Dutch pilot retrieval projects, the REACH (REtrieval And cure of Chronic Hepatitis C) study aimed to bring LFU patients with HCV back into care, describing that over the past 15 years up to 14% of all individuals with HCV were LFU and eligible for retrieval [5–8]. The REACH-study achieved the highest retrieval yield with 28% of all local LFU patients with HCV traced but this final turnout was still moderate at most. This may partially be explained by the abundant healthcare access barriers that occur in HCV risk groups and have extensively been described in both quantitative and qualitative studies [9–14]. These barriers include unstable housing, limited knowledge on HCV, perceived stigma by patients with HCV, lack of financial resources, competing (health) priorities, concomitant psychiatric disease, injection drug use, insufficient physician awareness and lack of referral. The most important financial barrier has been overcome since DAAs are fully reimbursed by the mandatory Dutch health care insurance since November 2015 (with exception of the obligatory deductible excess).

Limited studies are still available on patients’ perceived reasons for loss to follow-up and more importantly, the underlying processes have not been elucidated. Understanding why some patients stop attending the HCV outpatient clinic is essential to effectively target this LFU population with HCV and also to improve retention-in-care. This is of particular importance in the near future when patients with HCV with good healthcare access will all have been cured and the LFU population with HCV will increasingly exist of hard-to-reach patients for whom engagement strategies remain unclear.

The aim of this qualitative study is to explore reasons for previous loss to follow-up from a patients’ perspective in patients with HCV who have been brought back into care through the REACH-project. Furthermore, we aim to identify the key strategies that can be adopted in order to facilitate linkage to care in the remaining hard-to-reach population with HCV and contribute to future HCV elimination.

## Methods

### Study population

Between March through December 2017, patients with HCV who were evaluated at the Infectious diseases outpatient clinic of the University Medical Center Utrecht (UMCU) as part of the REACH-project [5] were sampled conveniently. Sampling stopped after theoretical saturation was achieved, which was defined as the moment in the analysis when no new insights or knowledge for the developed theory was gained through new research [15]. In short, the REACH-project aimed to retrieve and treat all LFU patients with HCV in the Utrecht region. Positive HCV diagnostics from the past were screened and linked to clinical records. Those patients who were LFU and eligible for retrieval were invited to attend the outpatient clinic for reevaluation. Patients were considered LFU when no follow-up appointment was scheduled at any hepatitis treatment center. Patients were contacted by mail and received a reminder after a period of four weeks and, if possible, were contacted by phone two weeks thereafter. Exclusion criteria for this study were: age<18 years and insufficient understanding of the Dutch language. Through this outreach attempt, the REACH-project traced 42 LFU patients with HCV. Of these retrieved patients, 3 patients could not take part in the interviews due to language barriers. A total of 28 individuals were invited to participate in the current study before theoretical saturation was achieved and 15 individuals were finally included in this study. The reasons for refusal comprised of: memory defects (N = 2), too emotional (N = 2) and not otherwise specified (N = 9).

### Data collection

Face-to-face semi-structured in-depth interviews were conducted to elucidate the (main) reason for previous loss to follow-up and to clarify experiences, perceptions and attitudes towards HCV care and therapy. Conversations were guided by a pre-defined topic list (S1 Table). All qualitative interviews opened with the same introductory question: ‘What do you think is the reason that you have not been visiting the outpatient clinic of a hepatitis treatment center for your hepatitis C?’ The average interview duration was 30 minutes. If possible, interviews were combined with regular (reevaluation) outpatient visits at the Infectious diseases department in order to maximize the participation rate in this hard-to-reach group. Interviews were conducted by one female researcher of the study team (P.K.) who was initially supervised and trained by a health researcher specialized in qualitative research (S.V.).

### Data analysis

A qualitative approach based on the grounded theory [16] was adopted to explore reasons for previous loss to follow-up in LFU patients with HCV who have been brought back into clinical care. Digitally recorded interviews were transcribed verbatim and entered into the software program NVivo V.11 [17] to support the analysis process. The interview transcripts were systematically analyzed through a process of substantive and theoretical coding assisted by memos and visual aids such as tables and diagrams. Three consecutive coding phases were adopted: open, axial and selective. First, the interviews were read out in full and reread to grasp the details. Through initial open coding meaningful paragraphs were selected and coded and categories were named based on similarities. Related concepts were identified from the different codes through subsequent axial coding. Finally, selective coding integrated all categories within a core category which led to the formulation of our substantive theory. The analysis was performed by two researchers (P.K. & S.V.) who compared coding decisions and discussed any differences until consensus was reached. Theoretical saturation [18] was achieved through constant comparison of the designated codes and derived concepts until sufficient understanding of the emerged concepts and themes had been acquired. During the data analysis process, additional expert review was conducted by an Infectious diseases specialist (J.A.) to evaluate if the derived concepts were fitting with clinical practice. The verbatim quotations in the result section were translated into English for the purpose of this manuscript. The index of qualitative variation (IQV) [19] was calculated (0.00 = no variation, 1.00 = maximum variation) to measure the variability in the study sample in terms of gender, country of birth (Dutch vs. non-Dutch), fibrosis degree distribution, history of (I)DU and treatment experience (i.e. previous exposure to interferon).

### Ethical considerations

This study has been approved by the institutional review board of the University Medical Center Utrecht. Written informed consent was obtained from all study participants.

## Results

### Participant characteristics and background

Characteristics of the 15 participants are presented in Table 1. The mean age of the participants was 51 years (±SD 10.4) and the median loss to follow-up duration was 7 years (IQR 4–10 years). There was high diversity in the study sample in terms of gender, fibrosis degree distribution and treatment experience (IQV of 0.96, 0.92 and 0.78 resp.) and moderate variation when considering history of (I)DU and country of birth (IQV 0.64 for both). Those 13 patients who declined to participate were comparable with the interviewees in terms of age, gender, country of birth (Dutch vs. non-Dutch), history of (intravenous) drug use (IDU), fibrosis degree distribution and treatment experience (all p ≥ .05).

**Table 1:** Characteristics of interview participants.

Transcript	Gender	Age	Country of birth	Year of HCV diagnosis	History of (I)DU	PEG-IFN experienced	Fibrosis stage at the reevaluation*	HBV / HIV co-infection	Current substance use	HBV / HIV co-infection
1	Female	55	Netherlands	2007	Yes	No	F0-F1	No	Nicotine	No
2	Male	55	Netherlands	2001	Yes	Yes	F0-F1	No	Nicotine	No
3	Male	63	Netherlands	2008	No	No	F2	No	None	No
4	Female	33	Russia	2004	No	No	F0-F1	No	None	No
5	Female	24	Netherlands	2012	Yes	No	F0-F1	No	GHB	No
6	Male	55	Netherlands	2007	Yes	No	F2	No	Marihuana, nicotine	No
7	Male	52	Netherlands	2009	Yes	No	F3	No	Alcohol, marihuana	No
8	Female	53	Netherlands	2005	Yes	No	F2	No	Nicotine	No
9	Male	44	Netherlands	2008	Yes	Yes	F3	Cleared HBV	Alcohol, marihuana, nicotine	Past HBV infection
10	Male	53	Suriname	2007	Yes	No	F0-F1	No	Cocaine, heroin, marihuana, nicotine	No
11	Male	62	Netherlands	2014	Yes	No	F4	Cleared HBV	Heroin, nicotine	Past HBV infection
12	Female	51	Netherlands	2011	Yes	Yes	F4	No	None	No
13	Female	54	Netherlands	2014	No	No	F0-F1	No	None	No
14	Male	54	Iran	2013	Yes	No	F2	No	Cocaine, heroine, marihuana	No
15	Male	60	Netherlands	2006	Yes	Yes	F2	Cleared HBV	Nicotine	Past HBV infection

*According to the METAVIR scoring system. (I)DU, (intravenous) drug use, alcohol use comprises ≥ .5 liter per day.

Previous intravenous drug use had been the main mode of transmission (N = 12) in the study population and eight participants continued to use methadone replacement therapy. Some had been incarcerated or homeless and eight participants were currently unemployed, incapacitated or retired.

### Maintaining the achieved balance as the basic process to explain loss to follow-up

**Fig 1:**
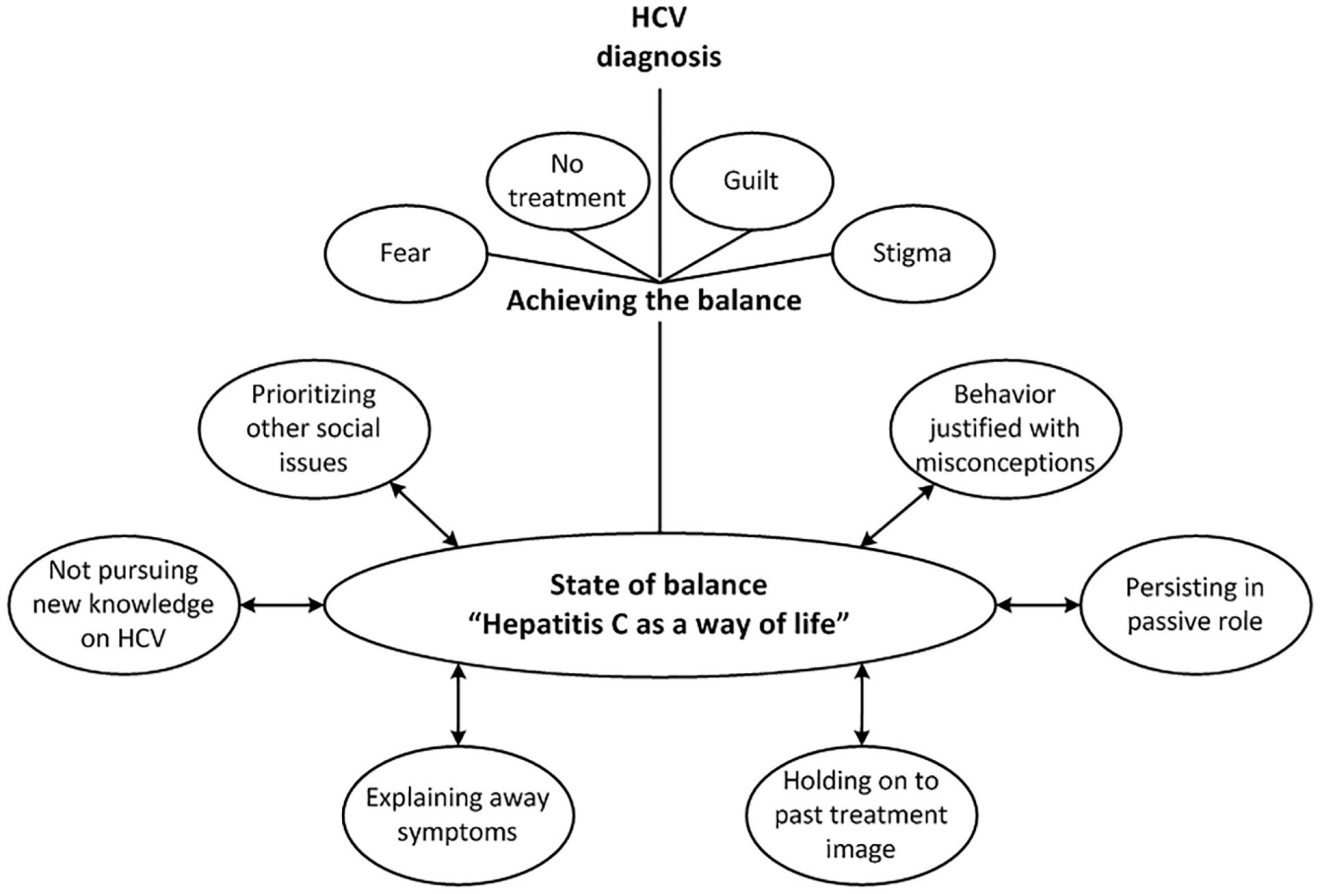
How lost to follow-up hepatitis C patients achieved and maintained a balancing perspective.

This study uncovered ‘maintaining the achieved balance’ as the basic socio-psychological process for patients with HCV who were lost to follow-up in our population (Fig 1). This ‘achieved balance’ is the result of the process that patients have gone through after initially having been diagnosed with chronic HCV. This achieved balance means that these patients, in a certain way, have come to terms with the fact that they are HCV-positive but want to keep it out of sight and in the margin of their lives. It is a state of relative comfort in which they do not want to act upon their chronic infection or be confronted with it. This stance elucidated why patients were lost to follow-up and further on, this attitude continued to manifest itself in the way that patients dealt with HCV related issues that crossed their path.

First, the process from the initial HCV diagnosis to the achieved balancing perspective is described. Second, the current practices that patients employ in order to maintain the achieved balance are outlined.

### From receiving the diagnosis to achieving a state of balance

Following the HCV diagnosis, patients went through a transformative process during which they compartmentalized their thoughts and feelings. Participants described initial emotional reactions upon receiving the HCV diagnosis such as “shock,” “fear” and thinking that “this can’t be right.” They experienced stigmatization and were confronted with feelings of guilt for having been at risk for the infection. In addition, they had to deal with disappointing treatment prospects. In each of these areas, the participants exhibited balance seeking behavior and made important choices in order to adapt to this life changing event and to finally achieve a new balance.

#### Dealing with stigma and feelings of guilt

From the beginning onwards, perceived stigma associated with the HCV infection vastly influenced the participants’ attitude towards the disease and they were subjected to negative opinions and prejudices, frequently from within their (personal) environment. Some spoke about people associating the liver infection with injecting drug use, HIV and homosexuality.

“Of course people are going to think: Whoa, hepatitis C, drug use, dangerous disease, we don’t want to… They associate it with HIV… because people know nothing about hepatitis C, B and A. That’s what they compare it with, because you are a drug user.”

Participants reacted to these preconceptions by keeping the infection hidden from the outside world. Usually only their most inner circle was informed about their HCV but also in this safe setting they still encountered judgment. By stowing the HCV far away in the back of their mind and by avoiding any confrontation with the disease, a relative peace of mind and sense of balance was eventually obtained. Feelings of guilt seemed to further contribute to their state of acquiescence. Some participants gave the impression to be struggling with these emotions thinking “I have done this to myself and should be glad to even be alive” and “It’s my own fault, so if there’s no drug then that’s that.” It motivated them to accept the consequences of the infection but it also reinforced their determination to bury their thoughts of HCV and move on. Both stigmatization and feelings of guilt thus constituted part of the reasons that the interviewees got out of touch with the healthcare system.

#### Believing no acceptable therapeutic options are available

Another major factor that accelerated the pace towards achieving a new balance included the (lack of) available therapy options. At a certain point, for many participants, treatment had been deferred for a variety of reasons including unstable social and living conditions, progressive liver disease and the prospect of the DAAs potentially becoming available. Not receiving treatment had been disappointing for some who said they “really wanted to receive therapy” or felt “shunted off” by their doctor. On the other hand, other participants had refused treatment because of the (unacceptable) side-effects and cure rates of pegylated-interferon (PEG-IFN) and consequently concluded there was no (tolerable) HCV therapy available: “I thought to myself: “I have got it and there is no drug.” Yeah, there is something but it will make you sick as a dog so…” Feeling forced to come to terms with such an undesirable situation greatly affected their disease management and propelled them into a balance where the HCV was marginalized.

Altogether, participants finally managed to deal with the life altering HCV diagnosis by creating a tolerable but also resistant balance life where the thought of HCV was kept firmly out of sight. After months or years of living with HCV, participants became accustomed to being chronically infected as “these feelings (of shock) wear out,” “the HCV has moved to the background” or emotions are “suppressed.”

#### Hepatitis C became a way of life with behavioral interventions

Over time, participants more or less acclimatized to the HCV and most of them made certain lifestyle adjustments that took their infectious state into account. For instance, concerns about transmitting HCV to their children, roommates and sexual partners prompted some interviewees to incorporate general preventative measures including keeping “razors and toothbrushes to myself” and preventing anyone to come in contact with their blood in case of cuts and wounds. In addition, participants attempted to negate the detrimental effects of the chronic infection through optimization of their general health and liver condition by “reading how to keep the liver healthy”, “refraining from alcohol and drugs,” “exercising and eating healthy” and also by taking homeopathic remedies. These precautions became a way of life and brought about a (false) sense of comfort in which they need not worry about the HCV.

### Maintaining the achieved balance

In spite of their clear comprehension of the possible repercussions of chronic HCV, participants seemed adamant about maintaining their relatively tranquil state of balance. They realized that the HCV “eats away your liver” and “prevents the blood from being purified.” In addition, the HCV infection was perceived as “a ticking time bomb” and some interviewees “knew people who had died from it.” Even so, the participants exhibited a wide variety of evasive behavior to avoid confrontation with the HCV including: prioritizing other social issues, clinging to past treatment images, misuse of misconceptions to justify behavior, explaining away signs and symptoms and persisting in the passive patient role. These different conducts were all directed at keeping the balance in their daily life by consciously sidelining the HCV.

#### Prioritizing other social issues

Many participants deprioritized the HCV below their other social responsibilities in order to keep the infection muffled away and to sustain the state of balance. They spoke about various matters that dictated their everyday life and overshadowed having chronic HCV. The topics that they referred to included having multiple young children, managing ongoing dependent drug use and having several comorbidities which caused a more substantial burden of disease compared to the HCV. The HCV was made subordinate to these issues and put on hold so the balance would be intact.

#### Image of past treatment determines current ideas about treatment

A negative image of the available treatment and the necessary investigations for HCV prevailed among the participants. They persisted in this belief which facilitated them staying in their undisturbed balancing state. Even though only two interviewees had previously received PEG-IFN therapy, the majority was well informed about the side-effects, either through their physicians or by peers who had received treatment. They also knew what chance of cure to expect from PEG-IFN treatment. Some had refused PEG-IFN therapy in the past:

“I know someone who got interferon and it was a bad experience for him. Like mood swings. And it affects your fitness of course and maybe also your immune system. It is a kill or cure drug really and I wasn’t up for that in my situation.”

Three participants who underwent a liver biopsy experienced this as quite painful and said it prevented them from coming back out of fear of having to repeat the procedure. A liver biopsy constitutes an invasive diagnostic procedure and for long has been the method of choice to determine the stage of liver fibrosis in patients with HCV.

“That is when I checked out. It was really unpleasant, that was no joke. I really thought… well, I had two good liver biopsies but the last one was just terrible. I don’t know how it happened but I didn’t want to do that anymore.”

The cumulative effect of such an unpleasant experience in addition to the negative image of PEG-IFN therapy made participants more determined to maintain the balance and avoid a new confrontation with their disease.

#### Behavior is justified through misconceptions and by not pursuing new knowledge on hepatitis C

Overall knowledge on HCV was low among the interviewees and misconceptions concerning the infection were common. Several of these misconceptions downgraded the severity of chronic HCV and made it less important, which helped participants to maintain the balance. This was a recurrent pattern that was observed. Some individuals were under the assumption that they had cleared the chronic infection in the past but still came to the outpatient clinic to affirm this notion. Occasionally, their general practitioner had confirmed this particular misconception. Another participant believed that his HCV was not “active” so he could not transmit it to others. This notion legitimized the participant not reaching out for care.

“I think my hepatitis C is not active so I believe I cannot transmit it to others. But I am not completely sure if that’s the case.”

The majority seldom actively sought updated information about HCV treatment probabilities and most were uninformed that novelties were to be expected. In addition, some did already learn about new HCV drugs with a shorter treatment course being available but had not looked into it deeper or followed-up on this information. In this manner, participants protected their balance and also avoided (new) disappointments. Again, misconceptions regarding the DAAs among the participants reinforced their stance as they thought it was all still “experimental” or that it was not available for their type of HCV.

#### Explaining away possible signs and symptoms and persisting in the passive patient role

Participants were familiar with the predominantly asymptomatic course of chronic HCV that could culminate in a notorious symptomatic end phase. Indeed, they frequently reported feeling physically well and denied having any HCV related symptoms at the time of the interview. However, whenever any (physical) complaints occurred in the past, participants often preferred to explain away the signs in order to safeguard their uncomplicated balance. Non-specific symptoms such as “fatigue” or a “general feeling of being unwell” were mostly assigned to other causes including “ageing,” “lack of sleep or exercise,” or “stress.” Some participants felt supported in this stance since they were repeatedly told everything was fine and that “the HCV was at rest or sleeping” during previous check-ups. For this reason, these outpatient visits were sometimes experienced as superfluous. Worries about the chronic infection could thus easily be shrugged off and the balance would be secured. Some participants indicated that they would be alarmed by obvious signs of disease progression including “yellow eyes” and “changed stool” but also realized that these symptoms indicated that the disease had reached an end stage. Still, they preferred to sustain the balance by sidetracking the HCV and not visiting the outpatient clinic while these signs were still absent. In the same way, participants would reason away the concerns from their family and friends:

“I think it was because of her that I had the previous investigations done. But in the end I will do what I want. She asked me if I didn’t need to visit the doctor for check-ups anymore. And I always said I would get to it”

Many interviewees had taken on a passive patient role and a recurrent explanation for not having attended the hepatology / infectious diseases outpatient clinic involved them awaiting a call from their physician. Not receiving notice from their doctor was interpreted as a reassuring sign. They did not take matters into their own hands but favored the comfortable balancing state instead. Others had been requested to make a follow-up appointment themselves within a couple of years or were encouraged to make inquiries about new therapy options. Participants however admitted that they probably would not have sought contact themselves had it not been for the reevaluation invitation letter.

## Discussion

When widespread implementation of universal DAA access has been realized, it will become increasingly important to identify and treat the LFU patients with HCV in order to achieve final elimination [5]. The LFU population with HCV can only efficiently be targeted if reasons for loss to follow-up and underlying processes are sufficiently understood. This study identified ‘maintaining the achieved balance’ as the basic socio-psychological process for LFU patients with HCV. Participants arrived at this balance through a transformative phase that followed the initial HCV diagnosis and they ended up marginalizing the HCV. The state of balance was shaped and influenced by different factors such as the natural history of HCV, the mode of transmission and the availability and burden of treatment. In the Dutch setting, with excellent access to care and DAA therapy, financial costs did not emerge as an influencing component but this may well be an attributing factor to loss to follow-up in patients with HCV in low resource regions. The balancing perspective was defended further along the road by repeated evasive behavioral patterns such as downplaying natural history of the disease or positively interpreting doctors’ messages. We hypothesize that the balance will be more unwavering in those patients who refused reevaluation (5%) and those who did not respond to the reevaluation request (57%) in the REACH-project [5]. Health care employees involved in HCV care may need to go the extra mile and employ additional targeted interventions in order to bring these hard-to-reach LFU patients with HCV back into care. Educating both the HCV infected population as well as the treating physicians on the improved diagnostic and treatment options for HCV appears essential as many of the behavioral patterns in our LFU HCV participants such as ‘explaining away symptoms’, ‘holding on to past treatment image’, ‘behavior justified with misconceptions’ and ‘not pursuing new knowledge on HCV’ appear linked with insufficient knowledge on these topics. A decentralized model of HCV care that facilitates HCV treatment uptake in primary and addiction healthcare settings could further enhance HCV care engagement in the hard-to-reach (LFU) HCV patients. The optimal approach to get LFU patients with HCV out of their steadfast stance remains to be investigated. Strategies may be aimed at improving the patients’ disease and treatment knowledge while removing misconceptions. In addition, outreach programs may be utilized to target and engage individuals who remain in the passive patient role.

Prior studies in LFU patients with HCV were predominantly of quantitative nature and aimed at identifying independent predictors for loss to follow-up (e.g. a history of substance abuse, psychiatric illness, undocumented liver fibrosis stage and absence of a life partner) [20–22], but seldom analyzed the patients’ perspective on the subject. Balkhy et al. performed a phone survey in LFU patients with HCV and hepatitis B virus (HBV) during which the far majority indicated that they were unaware that a follow-up appointment had been scheduled (69%) or declared to be uninformed about the need for follow-up (15%) [23]. The current grounded theory-based qualitative study is the first to clarify the underlying process and to describe the balancing perspective in LFU patients with HCV. A balancing mindset had been previously described in the overall population with HCV in the qualitative study of Faye et al. who reported on patients with a HCV infection passing through a changing stage after which they proceeded to the consolidating balancing perspective [24]. This process was set in motion in response to the overall feeling of “being condemned” by the HCV diagnosis which was accompanied by a consequential reactive depression. The current qualitative study confirms the existence of a state of balance in patients with HCV but rather than defending against HCV condemnation it was employed to consciously sidetrack the infection in this specific population with HCV that was LFU. The behavioral response in the current study population might best be appreciated in light of Lazarus and Folkman’s (1984) [25] transactional theory of stress and coping. First of all, participants readily labeled the HCV as potentially harmful after their initial appraisal of the diagnosis. Next, the lack of acceptable therapeutic options prompted many to conclude that resources to tackle this disease were unavailable. Feelings of lack of control more easily induce an ineffective emotion-focused coping response [26] which can also be observed in this specific patient group. Participants turned to different emotion-focused coping strategies such as exercising self-control (i.e. adapting life-style to limit detrimental effects of HCV), escape-avoidance (e.g. persisting in the passive patient role, explaining away signs and symptoms) and positive reappraisal (“the HCV is sleeping”). These coping strategies finally led to the conclusive act of sidelining the disease and thus becoming lost to follow-up.

The main strength of this study is the robust analysis that included double-coding, verbatim quotes and expert review of the outcomes to increase the reliability of the results. The findings of this study are applicable to settings with highly accessible HCV care and to patients with HCV who have been diagnosed in the PEG-IFN era. There were also some limitations to this study. The interviews were conducted after the participants had been brought back into care, which may have introduced recall bias. In order to maximize the participation rate, interviews were conducted by the physician who was responsible for the HCV reevaluation and consequential social desirability bias cannot be ruled out. Last, solely patients with HCV who were brought back into care were interviewed for the purpose of this study and they were inevitable recruited through convenience sampling. Both a purposeful sampling approach and an extended inclusion of those patients with HCV who refused participation in the REACH project and remained LFU could have increased the transferability of the results.

## Conclusion

The balancing perspective gives insight into why patients with HCV were not retained in care but also why they remained LFU thereafter. It has important implications for clinical practice as physicians should realize that this mindset can be persistent and that repeated efforts may be needed to finally trace and retrieve these patients.

## Supporting information

S1 Table Interview guide. (DOCX)

S2 Table Consolidated criteria for reporting qualitative studies (COREQ): 32-item checklist. (DOCX)

## Abbreviations

DAA: direct acting antivirals
HCV: hepatitis C virus
IDU: intravenous drug use
IQV: index of qualitative variation
LFU: lost to follow-up
PEG-IFN: pegylated-interferon
REACH: REtrieval And cure of Chronic Hepatitis C
UMCU: university medical center Utrecht
WHO: world health organization
