# Supplementary material for: The balancing perspective of hard-to-reach hepatitis C patients who were lost to follow-up: A qualitative study
Source: PLoS One. 2020 Apr 13;15(4):e0230756. doi: 10.1371/journal.pone.0230756 (PMC7153871; doi:10.1371/journal.pone.0230756)
Supplement: S1 Table — (DOCX) [file pone.0230756.s001.docx]

# S1 Interview guide

| **Topics** | **Questions (examples)** |
| --- | --- |
| General | Standard opening question: What do you think is the reason that you have not been visiting the outpatient clinic of a hepatitis treatment center for your hepatitis C? |
| Hepatitis C knowledge | Can you tell me what do you know about hepatitis C?  What do you know about hepatitis C transmission?  Do you know how you got infected with hepatitis C?  What do you know about hepatitis C treatment? |
| Hepatitis C treatment experience | How did you experience your previous hepatitis C treatment?  Did you experience any difficulties in taking the hepatitis C treatment?  What were your expectations about the hepatitis C treatment beforehand?  How was the relation with the health care professionals involved in your hepatitis C treatment? |
| Psychosocial | Can you tell me whether having hepatitis C has impacted your life?  Has having hepatitis C impacted your relationships?  What do other people think about hepatitis C, in your view / experience?  Have you experienced social support with regards to the hepatitis C infection? |
| Overall wellbeing | What do you think about your general health  Have you ever had health issues?  Have you ever used or abused drugs?  What do you think about your overall wellbeing? |
