# Supplementary material for: The balancing perspective of hard-to-reach hepatitis C patients who were lost to follow-up: A qualitative study
Source: PLoS One. 2020 Apr 13;15(4):e0230756. doi: 10.1371/journal.pone.0230756 (PMC7153871; doi:10.1371/journal.pone.0230756)
Supplement: S2 Table — (DOCX) [file pone.0230756.s002.docx]

**S2. Consolidated criteria for reporting qualitative studies (COREQ): 32-item checklist.**

Adapted from: Tong A, Sainsbury P, Craig J. Consolidated criteria for reporting qualitative research (COREQ): a 32-item checklist for interviews and focus groups. International Journal for Quality in Health Care. 2007; 19 (6): 349-57.

| **No. Item** | **Guide questions/description** | **Comment** |
| --- | --- | --- |
| **Domain 1: Research team and reflexivity** | | |
| *Personal Characteristics* | | |
| 1. Interviewer/facilitator | Which author/s conducted the interview or focus group? | Reported in manuscript |
| 2. Credentials | What were the researcher’s credentials? | Reported in manuscript |
| 3. Occupation | What was their occupation at the time of the study? | Reported in manuscript |
| 4. Gender | Was the researcher male or female? | Reported in manuscript |
| 5. Experience and training | What experience or training did the researcher have? | Reported in manuscript (training by experienced senior researcher) |
| *Relationship with participants* | | |
| 6. Relationship established | Was a relationship established prior to study commencement? | Reported in manuscript |
| 7. Participant knowledge of the interviewer | What did the participants know about the researcher? | Reported in manuscript |
| 8. Interviewer characteristics | What characteristics were reported about the interviewer/facilitator? | Reported in manuscript |
| **Domain 2: study design** | | |
| *Theoretical framework* | | |
| 9. Methodological orientation and Theory | What methodological orientation was stated to underpin the study? | Reported in manuscript |
| *Participant selection* | | |
| 10. Sampling | How were participants selected? | Reported in manuscript |
| 11. Method of approach | How were participants approached? | Reported in manuscript |
| 12. Sample size | How many participants were in the study? | Reported in manuscript |
| 13. Non-participation No none participants | How many people refused to participate or dropped out? Reasons? | Reported in manuscript |
| *Setting* | | |
| 14. Setting of data collection | Where was the data collected? | Reported in manuscript |
| 15. Presence of non-participants | Was anyone else present besides the participants and researchers? | Occasionally a family member |
| 16. Description of sample | What are the important characteristics of the sample? | Reported in manuscript |
| *Data collection* | | |
| 17. Interview guide | Were questions, prompts, guides provided by the authors? Was it pilot tested? | Interview guide reported in manuscript. Not pilot tested |
| 18. Repeat interviews | Were repeat inter views carried out? | No |
| 19. Audio/visual recording | Did the research use audio or visual  recording to collect the data? | Reported in manuscript |
| 20. Field notes | Were field notes made during and/or after the interview or focus group? | Reported in manuscript |
| 21. Duration | What was the duration of the inter views or focus group? | Reported in manuscript |
| 22. Data saturation | Was data saturation discussed? | Reported in manuscript |
| 23. Transcripts returned | Were transcripts returned to participants for comment and/or correction? | No, because of burden to participants |
| **Domain 3: analysis and findings** | | |
| *Data analysis* | | |
| 24. Number of data coders | How many data coders coded the data? | Reported in manuscript |
| 25. Description of the coding tree | Did authors provide a description of the coding tree? | No |
| 26. Derivation of themes | Were themes identified in advance or derived from the data? | Reported in manuscript |
| 27. Software What software | What software, if applicable, was used to manage the data? | Reported in manuscript |
| 28. Participant checking | Did participants provide feedback on the findings? | No, because of burden to participants |
| *Reporting* | | |
| 29. Quotations presented | Were participant quotations presented to illustrate the themes/findings? Was each quotation identified? | Reported in manuscript |
| 30. Data and findings consistent | Was there consistency between the data presented and the findings? | Reported in manuscript |
| 31. Clarity of major themes | Were major themes clearly presented in the findings? | Reported in manuscript |
| 32. Clarity of minor themes | Is there a description of diverse cases or discussion of minor themes? | Reported in manuscript |
